# GEICO (Spanish Group for Investigation on Ovarian Cancer) treatment guidelines in ovarian cancer 2012

**DOI:** 10.1007/s12094-012-0995-8

**Published:** 2013-03-07

**Authors:** A. González Martín, A. Redondo, M. Jurado, A. De Juan, I. Romero, I. Bover, J. M. Del Campo, A. Cervantes, Y. García, J. A. López-Guerrero, C. Mendiola, J. Palacios, M. J. Rubio, A. Poveda Velasco

**Affiliations:** 1 Medical Oncology Department, MD Anderson Cancer Center, C/Arturo Soria, 270, 28033 Madrid, Spain; 2 Medical Oncology Department, University Hospital La Paz, Madrid, Spain; 3 Department of Gynecology, School of Medicine, University of Navarra, Pamplona, Spain; 4 Department of Medical Oncology, University Hospital Marqués de Valdecilla, Santander, Spain; 5 Department of Medical Oncology, Fundación Instituto Valenciano de Oncología, Valencia, Spain; 6 Medical Oncology Department, Hospital Son Llàtzer, Palma de Mallorca, Islas Baleares, Spain; 7 Department of Medical Oncology, Vall d’Hebron University Hospital, Barcelona, Spain; 8 Department of Hematology and Medical Oncology, Institute of Health Research INCLIVA, Hospital Clínico Universitario, University of Valencia, Valencia, Spain; 9 Department of Medical Oncology, Sabadell Hospital, Parc Taulí Healthcare Corporation, Sabadell, Barcelona, Spain; 10 Laboratory of Molecular Biology, Fundación Instituto Valenciano de Oncología, Valencia, Spain; 11 Medical Oncology Department, University Hospital 12 de Octubre, Madrid, Spain; 12 Department of Pathology, University Hospital Ramón y Cajal, Madrid, Spain; 13 Department of Medical Oncology, University Hospital Reina Sofía, Córdoba, Spain; 14 Department of Medical Oncology, Fundación Instituto Valenciano de Oncología, Valencia, Spain

**Keywords:** Ovarian cancer, Treatment guidelines, Chemotherapy, Surgery, First line, Recurrent disease

## Abstract

In 2006, under the auspices of The Spanish Research Group for Ovarian Cancer (Spanish initials GEICO), the first “Treatment Guidelines in Ovarian Cancer” were developed and then published in Clinical and Translational Oncology by Poveda Velasco et al. (Clin Transl Oncol 9(5):308–316, 2007). Almost 6 years have elapsed and over this time, we have seen some important developments in the treatment of ovarian cancer. Significant changes were also introduced after the GCIG-sponsored 4th Consensus Conference on Ovarian Cancer by Stuart et al. (Int J Gynecol Cancer 21:750–755, 2011). So we decided to update the treatment guidelines in ovarian cancer and, with this objective, a group of investigators of the GEICO group met in February 2012. This study summarizes the presentations, discussions and evidence that were reviewed during the meeting and during further discussions of the manuscript.

## Methodology

During one and a half-day meeting, several topics on the management of ovarian cancer (OC) were reviewed by a panel of experts from GEICO about different areas. Each presentation covered the most accurate evidence about the specific topic and it was followed by a discussion in the panel of experts. The topics that were reviewed included: diagnosis, screening hereditary ovarian cancer, pathology, molecular biology, surgery of initial and advanced stages, systemic therapy of early and advanced stages, therapy of recurrent disease and strategies for the future in OC management.

To assign a level of evidence and a grade of recommendation to the different statements of this treatment guideline, it was decided to use the Infectious Diseases Society of America-US Public Health Service Grading System for ranking recommendations in clinical guidelines to determine the quality of evidence and strength of recommendation in each of the consensus recommendations (Table 1) [3].

**Table 1:** Infectious Diseases Society of America-US Public Health Service Grading System for ranking recommendations in clinical guidelines [3]

Category, grade	Definition
Strength of recommendation	
A	Good evidence to support a recommendation for use
B	Moderate evidence to support a recommendation for use
C	Poor evidence to support a recommendation
D	Moderate evidence to support a recommendation against use
E	Good evidence to support a recommendation against use
Quality of evidence	
I	Evidence from ≥1 properly randomized, controlled trial
II	Evidence from ≥1 well-designed clinical trial, without randomization; from cohort or case-controlled analytic studies (preferably from >1 center); from multiple time series; or from dramatic results from uncontrolled experiments
III	Evidence from opinions of respected authorities, based on clinical experience, descriptive studies, or reports of expert committees

When no unanimous consensus was achieved about the level of recommendation, an explanation of the different arguments was included in the manuscript.

Finally, a draft of the treatment guideline was sent to all the participants and also to other GEICO members for revision, discussion and final approval.

## Diagnosis

### Patients suspected to have adnexal mass

Transvaginal ultrasound (US) is considered the first-line imaging technique to be performed and includes morphology and color Doppler mapping. This will help determine its site of origin and characterize it as potentially benign or malignant. Transvaginal US has a high negative predictive value and is an excellent tool for ruling out OC [Quality of evidence, strength of recommendation: II, B] [4, 5].

CA 125, although not specific for EOC, is the most frequently used tumoral marker in the diagnostic process of an ovarian mass. It is elevated in 83 % of women with EOC but in only 50 % of those with stage I disease. In the presence of carcinomatosis, a proportion of CA 125/CEA >25 suggest ovarian cancer origin, and the opposite result, intestinal tumor. In young woman (<35 years), additional tumor markers like inhibin, AFP or B-hCG, should be measured if clinically indicated [3, 6].

It has been suggested that serum HE4 and CA 125 along with the algorithm ROMA (risk of ovarian malignancy) may be useful for determining whether a pelvic mass is malignant or benign. Nevertheless, a recently published study found that subjective assessment by US performed better than the ROMA and RMI (risk of malignancy index) in discriminating malignant from benign masses [7].

### Patients with suspected ovarian cancer

Surgical staging is the gold standard in OC and cannot be replaced by imaging techniques particularly in the detection of small peritoneal deposits. However, there is a trend toward increased use of imaging prior to surgical staging and cytoreduction to plan the surgical approach [8].

The staging accuracy of computed tomography (CT) or magnetic resonance imaging (MRI) is reported to be 70–90 %, although few studies comparing the accuracy of both techniques are available. The accuracy of detection of peritoneal implants with both CT and MRI is dependent on their location, size and the presence of ascites. Contrast-enhanced CT is the imaging modality of choice for staging of ovarian cancer, with the MRI being used as a problem-solving tool. In stages III and IV, the use of US is not recommended as staging modality due to its lower sensitivity to detect peritoneal metastases and other sites of disease compared to CT and MRI. CT can also be used to define disease extent that may help to evaluate the suitability for upfront cytoreductive surgery or for neoadjuvant chemotherapy. Although criteria for non-resectability vary widely between institutions and individual surgeons expertise, there are some preoperative imaging indicators, like the following: tumor deposits greater than 2 cm in the porta hepatis, diaphragmatic deposits, disease in the intersegmental fissure of the liver, lesser sac, small bowel mesentery and gastrosplenic ligament, parenchymal hepatic disease, and suprarenal aortic lymphadenopathy. However, this is highly dependent on the skills of the surgeon and the extension of the tumor. For instance, one solitary intraparenchymal liver metastasis can be potentially resected, and a diaphragmatic implant can be resected by well-trained surgeons. This fact and increasing surgical expertise in cytoreduction imply that preoperative CT predictors should be used with caution when assessing feasibility of primary cytoreduction [7].

Image-guided biopsy can be performed under US or CT guidance if, on the basis of imaging, the patient would benefit from neoadjuvant chemotherapy as histological confirmation of OC is mandatory. It is also essential prior to surgery if there is some clinical concern about the primary origin of the disease [7].

If there is uncertainty in the staging by radiological techniques, laparoscopic evaluation to select patients to cytoreductive surgery would play a great role in identifying patients unsuitable for optimal resection. Tissue for definitive histological diagnosis can also be obtained at the time of this procedure [III, B] [9].

Positron emission tomography using fluorodeoxyglucose (FDG-PET/CT) as staging tool in newly diagnosed advanced stages has not yet been fully determined. However, it provides an accurate assessment of disease in areas difficult to assess for metastases by CT and MRI like mediastinum, supraclavicular region, or small peritoneal implants. Normal-sized aortic lymph nodes with malignant involvement may also be identified by PET–CT [7].

Gastrointestinal (GI) workup in patients with diffuse carcinomatosis and GI symptoms may be indicated, including upper and/or lower endoscopy.

Since breast cancers can metastasize to the ovaries, more frequently when there is a bilateral involvement, mammography can help rule out this possibility and should be included in the preoperative workup for women older than 40 years who have not had one in the preceding 6–12 months.

Chest imaging, tumor markers, complete blood count and chemistry profile with liver and renal function are also part of the preoperative workup.

### Screening/early detection of ovarian cancer

When the disease is detected early, the 5-year survival is in excess of 90 % and this constitutes the rationale for the premise that detecting the disease in early stage may affect long-term survival. Although it has been shown that screening can detect the OC earlier and provide a survival benefit in the screening group, there is limited evidence that this can affect mortality from the disease and published data about it are conflicting [5].

In the largest trial UKTOCS using sequential CA 125 and transvaginal US, a survival advantage was achieved in the screened population compared with the control group. It was the consequence of a stage shift (82 % of screen-detected cancers were early stage compared with 34 % of those from the control group, *p* < 0.0001). Final results of this study are awaited in 2015 before definitive conclusions can be drawn [10, 11].

On the contrary, the PLCO study reported no mortality benefit with OC screening, although some concerns have been raised about trial design [12]. Despite the generalized belief that OC lacks obvious warning symptoms, a recent review of a large number of publications suggest that up to 90 % of women experience symptoms before their diagnosis. A symptom index has been developed and when combined with CA 125 and HE4 showed an increase in specificity to 98 %.

Among high-risk women (mainly mutations in the BRCA1/2 genes), the sensitivity and effectiveness of screening are yet to be established. Several trials are still under way and their results will come out during the next years [13].

## Hereditary ovarian cancer

Approximately 13 % of EOCs are associated with inheritance of an autosomal dominant genetic aberration, which leads to cancer predisposition with a moderate to high penetrance [14]. BRCA1 and BRCA2 proteins are essential to the homologous recombination DNA repair mechanism, in recognizing double-strand breaks.

Currently, Dragon Database for Exploration of Ovarian Cancer Genes (DDOC) contains a set of 379 human genes experimentally verified as involved in OC [15]. Table 2 shows the best-known genes [16].

**Table 2:** Genes implicated in hereditary ovarian carcinoma

FA-BRCA pathway genes	HBOC
BRCA1	
BRCA2	
RAD51C	Low penetrance genes
RAD51D	
BRP1	
BARD1	
CHEK2	
MRE1 1A	
NBN	
PALB2	
RAD50	
Mismatch repair genes	Lynch syndrome
MLH1	
MSH2	
MSH6	
PMS2	
Other genes	Li–Fraumeni syndrome
TP53	

*HBOC* hereditary breast and ovarian cancer

The estimated lifetime risk is 1 case in every 70 women, which is a 1.4 % lifetime incidence [17]. This estimated lifetime risk increases to 3 % in a second-degree relative, to 5 % in a first-degree relative, and up to 9 % in Lynch syndrome (hereditary nonpolyposis colorectal cancer). It reaches 39–60 % in BRCA1 mutation and 11–30 % in BRCA2 mutation [18].

Oncologists have a crucial opportunity to utilize risk assessment and cancer prevention strategies to interrupt the initiation or progression of OC in cancer survivors and individuals at high risk of developing cancer [19]. The Amsterdam II criteria [20] and Revised Bethesda Guidelines [21] (Table 3) can be used to identify the criteria for referring a patient to the genetic counseling unit (GCU), based on three questions (Table 4). A detailed family history of cancer taken at the first visit with the oncology provider, and based on the following three questions, can raise the suspicion of a hereditary cancer syndrome and be referred to the GCU [II, A] [22].

**Table 3:** The Revised Bethesda Guidelines and Amsterdam II criteria [21, 25]

The Revised Bethesda Guidelines for testing colorectal tumors for microsatellite instability (MSI)
Tumors from individuals should be tested for MSI in the following situations: 1. Colorectal cancer diagnosed in a patient who is less than 50 years of age 2. Presence of synchronous, metachronous colorectal, or other HNPCC-associated tumors,^a^ regardless of age 3. Colorectal cancer with the MSI-H^b^ histology^c^ diagnosed in a patient who is less than 60 years of age^d^ 4. Colorectal cancer diagnosed in one or more first-degree relatives with an HNPCC-related tumor, with one of the cancers being diagnosed under age 50 years 5. Colorectal cancer diagnosed in two or more first- or second-degree relatives with HNPCC-related tumors, regardless of age
Amsterdam II Clinical criteria for families with Lynch syndrome
Each of the following criteria must be fulfilled: 3 or more relatives with an associated cancer (colorectal cancer, or cancer of the endometrium, small intestine, ureter or renal pelvis) 2 or more successive generations affected 1 or more relatives diagnosed before the age of 50 years 1 should be a first-degree relative of the other two Familial adenomatous polyposis (FAP) should be excluded in cases of colorectal carcinoma Tumors should be verified by pathologic examination

^a^Hereditary nonpolyposis colorectal cancer (HNPCC)-related tumors include colorectal, endometrial, stomach, ovarian, pancreas, ureter and renal pelvis, biliary tract, and brain (usually glioblastoma as seen in Turcot syndrome) tumors, sebaceous gland adenomas and keratoacanthomas in Muir–Torre syndrome, and carcinoma of the small bowel [26]

^b^MSI-H, microsatellite instability-high, in tumors refers to changes in two or more of the five National Cancer Institute recommended panels of microsatellite markers

^c^Presence of tumor infiltrating lymphocytes, Crohn’s-like lymphocytic reaction, mucinous/signet-ring differentiation, or medullary growth pattern

^d^There was no consensus among the Workshop participants on whether to include the age criteria in guideline 3 above; participants voted to keep less than 60 years of age in the guidelines

**Table 4:** Family history in three questions

Family history in three questions
1. How old was the diagnosis?
2. First-degree relatives
HBOC
LYNCH
3. Second-degree relative

*HBOC* hereditary breast and ovarian cancer

Two hereditary syndromes, namely hereditary breast and ovarian cancer (HBOC) and Lynch syndrome, with mutations in BRCA1/2 genes and mismatch repair genes, respectively, have been identified (Table 5).

**Table 5:** Mutations in BRCA1/2 genes and mismatch repair genes in HBOC and Lynch syndrome

	HBOC	Lynch syndrome
Genes	BRCA1 and BRCA2	MLH1, MSH2, MSH6 and PMS2
Increased risk of cancer	Breast, ovarian, pancreatic, prostate	Colon, uterine, ovarian, other cancers of the digestive tract

*HBOC* hereditary breast and ovarian cancer

Hereditary nonpolyposis colorectal cancer (HNPCC) or Lynch syndrome is a common, autosomal dominant syndrome characterized by early onset (average age at onset <45 years), the development of neoplastic lesions in a variety of tissues (colon, gastrointestinal tract, ovary, and uterus) and microsatellite instability (MSI). For carriers of Lynch syndrome, the estimated lifetime risk of OC is 9–12 %.

HBOC is characterized by an increased susceptibility to breast cancer occurring at a young age, bilateral breast cancer, male breast cancer, and OC at any age. Other cancers such as prostate cancer, pancreatic cancer, gastrointestinal cancers, melanoma and laryngeal cancer occur more frequently in HBOC families. Hereditary site-specific breast cancer families are characterized by early-onset breast cancer with or without male cases, but without ovarian cancer. For this consensus, both will be referred collectively as hereditary breast and/or ovarian cancer. Germline mutations in the BRCA1 and BRCA2 genes are responsible for cancer susceptibility in the majority of HBOC families.

There is a high rate of tubal intraepithelial carcinoma (TIC) in high-risk women undergoing risk-reducing salpingo-oophorectomy. Recent studies have documented that up to 59 % of high-grade pelvic (non-uterine) serous carcinomas are associated with serous TICs. This is consistent with the hypothesis that the fallopian tube is the source of a majority of these tumors [23]. Approximately 30 % of women with fallopian tube cancer have a mutation in BRCA1 or BRCA2 [24, 25]. In women with BRCA1 and BRCA2 mutations, the use of risk-reducing mastectomy was associated with a lower risk of breast cancer; risk-reducing salpingo-oophorectomy was associated with a lower risk of breast and ovarian cancer [32].

Patients with invasive EOC with a germline mutation in BRCA1 or BRCA2 were associated with improved 5-year overall survival. BRCA2 carriers had the best prognosis. This may be due to distinct clinical behavior and/or to a better response to chemotherapy.

Primary prevention by detecting more women at high risk for the disease development by applying new methods of prevention like risk-reducing surgery is perhaps a more useful strategy for reducing mortality for OC patients. As mentioned before, there is accumulating evidence suggesting that serous neoplasia originates in secretory fallopian tube surface epithelium. This intraepithelial lesion has been found in about 43 % of women with advanced serous cancers, so this finding may potentially serve as a carcinogenic marker. Its identification in women at uncertain risk by means of minimally invasive methods or with salpingectomy at the time of other major surgical procedures in women who have completed their childbearing may be a reasonable strategy that deserves investigation [26–28].

## Pathology and molecular genetics

Several studies have shown that OC is not a single disease, but instead is composed of a diverse group of tumors that can be classified based on distinctive morphologic and molecular genetics features [29]. Additionally, the various subtypes have a different natural behavior and prognosis [33].

Based on light microscopy and molecular genetics, they can be subdivided into at least five main subtypes [30], classified by cell type into serous, mucinous, endometrioid, clear cell, and Brenner (transitional) tumors corresponding to different types of epithelia in the organs of the female reproductive tract. Once grouped by cell type, the tumors can be further subdivided into those that are clearly benign (cystadenomas), those that are clearly malignant (carcinomas), and those that have features somewhere between these two, variably called “atypical proliferative” tumors, tumors of “low malignant potential” or tumors of “borderline” malignancy. These subtypes show differences in epidemiological and genetic risk factors, precursor lesions, spreading patterns, molecular events during oncogenesis, response to chemotherapy and outcome.

Histopathological findings strongly suggest that there is a morphological and biological spectrum which starts with a benign serous cystadenoma/adenofibroma, and continues from a proliferative tumor (atypical proliferative serous tumor) to a non-invasive carcinoma (non-invasive micropapillary serous carcinomas), ending with an invasive low-grade serous carcinoma (LGSC) (invasive micropapillary serous carcinomas). Type I tumors (low-grade serous carcinoma, mucinous carcinoma, endometrioid carcinoma, malignant Brenner tumor, and clear cell carcinoma) develop in a stepwise manner from well-recognized precursors, namely borderline tumors that in turn develop from cystadenomas and adenofibromas [31]. Type II tumors are high grade at presentation and are currently classified as high-grade serous carcinoma (HGSC), malignant mixed mesodermal tumors (carcinosarcomas), and undifferentiated carcinoma [32]. High-grade serous histology is more frequent in advanced stage. HGSC and LGSC have different histology, molecular genetic alterations and biology [33] (Table 6). HGSC displays TP53 mutations in over 90 % of cases and rarely harbors the mutations that are found in the type I tumors (KRAS and BRAF). They are also characterized by potential aberrations in BRCA1 and BRCA2, in up to 50 % of cases [34].

**Table 6:** High-grade serous carcinoma (HGSC) and low-grade serous carcinoma (LGSC) differences

	HGSC	LGSC
Risk factors	BRCA 1/2	?
Precursor lesions	Tubal intraepithelial carcinoma	Serous borderline tumor
Pattern of spread	Very early transcoelomic	Transcoelomic spread
Molecular abnormalities	BRCA, p53	BRAF, KRAS
Ki-67	High	Low
Chemosensitivity	High	Intermediate
Estrogen receptor	2/3	?
Prognosis	Poor	Intermediate
Median age at presentation	Higher	Lower

Mucinous borderline tumors are classified into two different clinicopathological types: intestinal (85 % of cases) and endocervical (15 %). Bilateralism, in the case of a mucinous tumor, suggests that the possibility of adenocarcinoma metastasis, generally of gastrointestinal or pancreatic origin, should be ruled out. Moreover, bilateral ovarian tumors accompanied by pseudomyxoma peritonei tend to be of appendicular origin [35]. Immunohistochemical study with cytokeratin 7 and 20 can help in defining the origin of the lesion (EOC: CK7+/CK20−; metastasis: CK7−/CK20+).

Clear cell carcinomas (CCCs) constitute a spectrum of tumors of differing degrees of malignancy which are characterized by being formed by clear hobnailed, eosinophilic cells. Adenofibromas and clear cell borderline tumors are very uncommon. Given that CCCs frequently show a mixture of growth patterns and nuclear atypia, they are tumors that do not tend to progress from a histological point of view [36].

In CCCs, molecular alterations similar to those of EOCs have been described, but with a different frequency: beta-catenin mutations (5 %), PTEN (5–8 %), K-RAS (15–30 %), and MSI (5 %) [37–39].

Ovarian clear cell and endometrioid carcinomas may stem from endometriosis. ARID1A mutations were observed in 55 of 119 ovarian CCCs (46 %), 10 of 33 endometrioid carcinomas (30 %), and none of 76 high-grade serous ovarian carcinomas [40].

Identifying patients who would benefit from particular targeted therapies is an important objective. The first step in developing tools to improve cancer control for OC is to recognize that OC represents many diseases [41]. Additionally, it is highly recommended that human biospecimens for translational studies be available from clinical trials.

## Surgical treatment

Surgery is the cornerstone in treatment of ovarian cancer. All patients with newly diagnosed disease who are fit for surgery should be considered for a full staging laparotomy for accurate information on disease and histology. This is important for predicting prognosis and decision of postsurgical therapy. Based on published improved outcomes, it is recommended that a gynecologic oncologist surgeon perform the primary surgery [II, A] [42]. Types of surgery for OC may include: primary surgery for staging and cytoreduction, interval debulking surgery (IDS); secondary cytoreduction, second look operation, and palliative surgery.

### Early disease (clinical stage I/II)

The aim was proper staging of disease and removal of all macroscopic tumor. Surgery can be performed either preferably by laparotomy, which is the most accepted procedure, or minimally invasive surgery in selected patients if performed by an experienced gynecologic oncologist. Procedures must comprise thorough inspection and palpation of all peritoneal surface, total hysterectomy and bilateral salpingo-oophorectomy (TH + BSO), omentectomy, pelvic and bilateral aortic lymphadenectomy up to the renal vessels, biopsies of pelvic peritoneum, paracolic gutters and right subdiaphragmatic area, sampling of ascites or peritoneal washing for cytology (when no ascites is found). Appendectomy is recommended in mucinous tumors [43].

Several updated studies have concluded that completeness of surgical staging in patients with early stage was significantly associated with better outcomes. The 2010 GCIG consensus stated that surgical staging should be mandatory and be performed by a gynecologic oncologist [II, A] [2]. Under-staged patients in previous surgery should be re-staged according to the same surgical principles mentioned above. The same principle should be applied for patients with poorly differentiated tumor, clear cell histology, and stage IC due to ovarian surface involvement [44].

For a young patient (<40 years) who wishes to maintain fertility, a unilateral salpingo-oophorectomy may be adequate for selected stage I tumors (Ia and Ic due to intraoperative rupture but with negative cytology, grade 1 or 2, but not stage IB) in addition to the staging procedure. After fulfilling their wishes of fertility, salpingo-oophorectomy is recommended [III, B]. The practice of carrying out a wedge biopsy on a grossly normal contralateral ovary should be discouraged. If the histology is of endometrioid type, an endometrial biopsy should be performed to rule out a concurrent endometrial cancer. Most studies have found that conservative treatment is suitable for patients with serous, mucinous, or endometrioid carcinoma but not for patients with high-risk factors such as clear cell or poorly differentiated carcinoma [45, 46].

### Advanced disease (III–IV)

The standard treatment of advanced OC is cytoreductive surgery followed by platinum-based combination chemotherapy. Although the ultimate goal is cytoreduction to microscopic disease by removing all visible disease, successful cytoreduction to small-volume disease (<1 cm) increases the frequency of complete response and overall survival [II, A] [47–49]. According to the 2010 OC Consensus conference held in Vancouver, the term “optimal” cytoreduction should be reserved for those with no macroscopic residual disease [2].

The maximal surgical effort may comprise sometimes the following procedures: TAH + BSO (supracervical hysterectomy is also appropriate in some circumstances), omentectomy, radical pelvic dissection, bowel resection, diaphragm or other peritoneal surface stripping, splenectomy, partial hepatectomy, partial gastrectomy or cystectomy, distal pancreatectomy, or lymphadenectomy (bulky or suspicious lymph nodes resection). If complete cytoreduction is achieved, lymphadenectomy may increase overall survival [55].

Some contraindications for the outcome of this “maximum” effort surgery have been pointed out such as the following: poor performance status (Karnofsky <40), mesentery root involvement, extra-abdominal visceral disease, multiple intraparenchymal liver metastases, or intestinal massive-serosal carcinomatosis [II, A]. Nevertheless, as surgery on advanced OC evolves, some of these contraindications are being overcome [50, 51].

Delayed primary surgery after neoadjuvant chemotherapy or IDS is an option for selected patients with stage IIIC and IV. Despite the results of a recent randomized controlled trial, this therapeutical plan remains controversial. According to some authors, neoadjuvant chemotherapy followed by IDS should be reserved for patients who do not have access to gynecologic or surgical oncologist, cannot tolerate the procedure, and/or for whom optimal cytoreduction is deemed not feasible by an experienced surgical team. The goal of this surgery should be the same as in primary surgery and comprise the same procedures if necessary [52, 56].

Patients with low volume (<1 cm) of residual disease after upfront primary debulking surgery are potential candidates for intraperitoneal (IP) chemotherapy and, in these patients, consideration should be given to placement of an IP catheter with initial surgery [53].

## Systemic therapy in first line

### Early stages

The results of studies published in the last 10 years support adjuvant treatment with chemotherapy after surgery in most patients showing early stages of epithelial ovarian cancer [54–58]. Only low-risk patients (stages IA/B Grade 1 and no clear cell histology) with correct surgical staging require observation exclusively, as long-term survival after surgery is above 90 % [59].

**Fig. 1:**
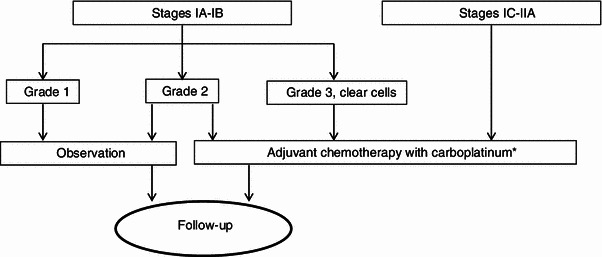
Adjuvant therapy in stage I and stage IIA epithelial ovarian cancer

Adjuvant treatment with chemotherapy after surgery is indicated in high-risk early stages (IA and IB Grade 3, clear cell tumors and any grade of stages IC and IIA) [I, A]. However, there is no consensus on the need to treat stages IA/B Grade 2. For these cases, both observation and adjuvant treatment can be regarded as valid options (Fig. 1) [1]. The ICON 1 [62] and ACTION [60] studies, as well as the combined analysis of both [61], support the use of adjuvant chemotherapy in early stages with a high risk of relapse, as an improvement is seen in both disease-free survival and overall survival (OS) when adjuvant platinum-based chemotherapy is given.

As yet, there are no data from comparative studies on these stages to determine the value of adding paclitaxel to platinum. Chemotherapy must at least include carboplatinum (AUC 5–7.5). The GOG-157 trial compared the administration of 3 cycles of paclitaxel (175 mg/m^2^ over 3 h) and carboplatin (AUC 7.5) versus 6 cycles of the same combination in patients with stage I optimally staged. The risk of recurrence was 33 % lower for patients treated with 6 cycles. However, this difference did not reach statistical significance (95 % CI 0.49–1.16). Additionally, the 6-cycle arm was associated to a higher hematological toxicity and neurological grade 2–4 toxicity (28 vs. 13 %). Based on this trial, the current standard chemotherapy in the adjuvant setting consists of at least 3 cycles of paclitaxel and carboplatin [64].

### Advanced stages

#### Conventional chemotherapy

The current therapeutic strategy generally recommended for the treatment of advanced OC (IIc–IV) is optimal cytoreductive surgery followed by 6 cycles of paclitaxel and carboplatin [61–63]. The recommendation to include paclitaxel with platinum was based on two large randomized studies that established the superiority of the paclitaxel–cisplatin combination versus cyclophosphamide–cisplatin [64, 65]. Mature data maintain this results [70], and the combined analysis showed survival benefits with the paclitaxel scheme [66]. Subsequently, three randomized studies that included over 1,500 patients compared paclitaxel–cisplatin with paclitaxel–carboplatin; no differences were found in response to progression-free survival (PFS), but toxicity and tolerability profiles were better for the carboplatin-combination arms [67–69]. According to the 4th Ovarian Cancer Consensus Conference [2], the standard treatment should include paclitaxel (175 mg/m^2^) and carboplatin (AUC 5–7.5) every 3 weeks for 6 cycles [I, A]. The data from the MITO-2 randomized, multicenter clinical trial conducted by the Multicenter Italian Trials in Ovarian (MITO) Cancer group indicate that the activity of carboplatin and pegylated liposomal doxorubicin (PLD) is similar to that of standard first-line therapy with carboplatin/paclitaxel in advanced ovarian cancer. Moreover, in terms of safety, the study group had a lower incidence of peripheral neuropathy (15 %) and alopecia (14 %) than the control group (47 and 63 %, respectively) [70]. However, this was a study with a design of superiority that did not meet its primary endpoint. For this reason, it should be considered a negative study that has not changed the standard of care. However, it can be considered a treatment option for patients not eligible to receive taxol.

#### Triplet and doublet chemotherapies

Various treatment strategies have been applied with a view to improving the prognosis of these patients. One is the addition of one or more drugs without cross resistance with the therapy which up to now has been considered standard (carboplatin–paclitaxel). Three studies have been published in this area evaluating the addition of a third drug in triple or sequential form (epirubicin, gemcitabine, topotecan or PLD) [71–73].

Unfortunately, none has been shown to be of any advantage. Adding one or more drugs only signifies greater toxicity. The administration of a third or more drugs is not therefore recommended at present [I, E].

#### Maintenance chemotherapy

In an effort to improve the modest results obtained in patients with suboptimal debulking, trials have been conducted with consolidation or maintenance chemotherapy. To date, none of the treatments administered following initial induction with platinum/paclitaxel have shown to improve survival [74–78].

#### Neoadjuvant chemotherapy (NAC)

A meta-analysis examined the effectiveness of NAC with platinum and IDS for advanced ovarian cancer, including 835 patients from 51 studies. Patients who had undergone IDS after an attempt at primary surgery were found to have survived for less time than those who had primary surgery. However, the review only included phases I to II and retrospective studies [79]. This observation was the background for a study conducted by the European Organisation for the Research and Treatment of Cancer (EORTC) Gynaecological Cancer Group, in conjunction with the National Cancer Institute of Canada (NCIC) Clinical Trials Group (EORTC-55971) between 1998 and 2006 that included 670 women with stages IIIC or IV ovarian cancer [80]. The women were randomized to primary debulking surgery, followed by at least six cycles of platinum-based chemotherapy or three cycles of NAC, also platinum based, followed by IDS, and by at least three more cycles of platinum-based chemotherapy. The median OS after primary debulking surgery was 29 months, compared to 30 months for the patients assigned to NAC. The hazard ratio for death in the group assigned to NAC followed by interval debulking, as compared with the group assigned to primary debulking surgery followed by chemotherapy, was 0.98. The subgroup of patients who had optimal debulking (<1 cm) after primary debulking surgery or NAC followed by IDS had the best median OS [92].

Despite these results, NAC is still a controversial issue. Some concerns have risen from the quality of the surgery performed in this trial and the wide use of NAC even in patient candidate for optimal upfront debulking surgery. In conclusion, NAC should be reserved for those who cannot tolerate PDS and/or for whom optimal cytoreduction is not feasible after an adequate evaluation performed by a surgical team well trained on cytoreduction [I, B] [59].

#### Dose-dense regimen

A Japanese study evaluated the weekly (dose-dense) administration of paclitaxel in patients with advanced ovarian cancer. This was a phase III study which included 631 patients with stage II–IV (82 % were stage III–IV) [81]. They were randomized to receive paclitaxel every 3 weeks at the dose of 185 mg/m^2^ versus weekly paclitaxel (dose-dense regimen) at 80 mg/m^2^ for 3 weeks. Both arms received carboplatin at an AUC of 6 every 3 weeks. According to the published results, there was a statistically significant improvement in PFS (28 vs. 17.2 months, *p* = 0.015) in favor of the dose-dense administration arm. After long-term follow-up, at 6.4 years of median, it continues to show a highly statistically significant improvement in median PFS in favor of the dd-TC group compared with the c-TC group [28.1 vs. 17.5 months, hazard ratio (HR) 0.75, 95 % CI 0.62–0.91, *p* = 0.0037], and also a benefit in the 5-year overall survival rate (58.7 vs. 51.1 %, HR 0.79, 95 % CI 0.63–0.99) [82] [I, B]. This interesting strategy should be confirmed in the Caucasian population, as the Japanese population may have genetic differences that could influence the pharmacokinetics or pharmacodynamics of the weekly schedule. In fact, toxicity in the Japanese population was significant with 36 % of patients discontinuing therapy due to side effects. Two European trials are dealing with this topic, MITO 7 and ICON 8, however, they have not been presented and we do not have definitive data for a formal recommendation of this schedule to Caucasian patients with advanced ovarian cancer.

#### Intraperitoneal chemotherapy (IP CT)

IP chemotherapy has certain clinical and pharmacological advantages over intravenous chemotherapy in patients with EOC limited to the abdominal cavity, who have had optimal debulking surgery. Three large randomized studies found improvements in PFS and OS (Table 7) [83, 84, 93].

**Table 7:** Summary of studies in intraperitoneal chemotherapy

Study	Control regimen	Experimental regimen	Eligible patients	No. of patients
SWOG 8501/GOG 104, Alberts et al. [83]	Cisplatin, 100 mg/m^2^ i.v.; cyclophosphamide, 600 mg/m^2^ i.v. q 3 weeks × 6	Cisplatin. 100 mg/m^2^ i.p.; cyclophosphamide, 600 mg/m^2^ i.v. q 3 weeks × 6	Stage III, ≤2 cm residual	546
GOG 114/SWOG 9227, Markman et al. [84]	Cisplatin. 75 mg/m^2^ i.v.; paclitaxel, 135 mg/m^2^ 24-h i.v. q 3 weeks × 6	Carboplatin, AUC 9 i.v. q 28 days × 2; cisplatin, 100 mg/m^2^ i.p.; paclitaxel, 135 mg/m^2^ 24 h i.v. q 3 weeks × 6	Stage III, ≤1 cm residual	462
GOG 172, Armstrong et al. [85]	Cisplatin, 75 mg/m^2^ i.v.; paclitaxel, 135 mg/m^2^ 24 h i.v. q 3 weeks × 6	Paclitaxel, 135 mg/m^2^ 24-h i.v.; Cisplatin, 100 mg/m^2^ i.p.; paclitaxel, 60 mg/m^2^ i.p. on day 8 q 3 weeks × 6	Stage III, ≤1 cm residual	415

The last and most important of them was the GOG-172 study, published in 2006 by Dr. Armstrong [85] that included 415 patients with stage III and residual tumor ≤1 cm. The patients were randomized to receive either cisplatin 75 mg/m^2^ i.v. plus paclitaxel 135 mg/m^2^ i.v. by continuous infusion over 24 h every 3 weeks for 6 cycles or paclitaxel 135 mg/m^2^ i.v. followed by cisplatin 100 mg/m^2^ i.p. plus paclitaxel 60 mg/m^2^ i.p. on day 8 every 3 weeks for 6 cycles. As with the previous study, patients in the IP CT arm had longer PFS (23.8 vs. 18.3 months, *p* = 0.05) and OS (65.6 vs. 49.7 months, *p* = 0.03).

A number of meta-analyses and systematic reviews have analyzed the above studies together and have categorically confirmed the results in terms of benefits over PFS and OS. However, what they also show is an increase in toxicity, especially fever, fatigue, gastrointestinal problems, infection, pain, deafness and metabolic and neurological abnormalities [86].

IP CT has therefore shown to be superior to i.v. CT and is another standard option in the management of patients with stage III and residual tumor ≤1 cm, even taking into account the technical issues with this method and the toxicity which at present limits its routine use [I, A]. Because of these difficulties, IP CT may be an option only for selected patients and selected centers.

#### Antiangiogenic therapy

Two phase III trials (GOG 0218 and ICON7) [87, 88] have shown that bevacizumab may be beneficial when added to standard chemotherapy with paclitaxel and carboplatin in the first-line treatment of ovarian cancer.

Although both trials explored the same concept, there were some differences in the design of the studies that it is worth to be explained. The GOG-218 was a randomized double blinded trial comparing bevacizumab with placebo in three different arms: control arm with placebo during chemotherapy followed by a maintenance phase with placebo, initiation group with bevacizumab added to chemotherapy followed by placebo and the throughout group with bevacizumab added to initial chemotherapy followed by a limited period of maintenance with bevacizumab. In the ICON-7, the design was simpler, without placebo and with only two arms, a control group with paclitaxel and carboplatin and the experimental arm with bevacizumab added to paclitaxel–carboplatin followed by a maintenance period with bevacizumab. There were also differences in the duration of bevacizumab (15 months in GOG-218 vs. 12 months in ICON-7), the dose (15 mg/kg in GOG-218 vs. 7.5 mg/kg in ICON 7) and the population of patients included (FIGO stage III–IV with macroscopic residual disease after surgery in GOG-218 vs. FIGO stage I of high risk to stage IV in ICON-7).

Both trials met their primary endpoint. In the COG-218, the administration of bevacizumab concurrently with chemotherapy followed by a maintenance phase of bevacizumab was associated with a significant increment in the median PFS from 10.3 to 14.1 months (HR 0.71, 95 % CI 0.625–0.824, *p* < 0.001). In the ICON-7, the median PFS was 17.3 months in the standard-therapy group and 19.0 months in the bevacizumab group (HR 0.81, 95 % CI 0.70–0.94, *p* = 0.004).

Regarding tolerability, the main toxicity associated to the administration of bevacizumab was hypertension, which was grade 2 or higher in 22.9 and 18.9 % of patients in the GOG-218 and ICON-7 trial, respectively. Moreover, there were no significant differences in the rates of other adverse events, including gastrointestinal perforation or fistula, proteinuria of grade 3 or greater, neutropenia of grade 4 or greater or febrile neutropenia, venous or arterial thrombosis, and wound disruption.

Some additional exploratory analysis of both trials has shown the following data:

1. A sensitive analysis of the GOG-218 censoring progression by CA 125 and considering only the patients who progressed by radiological imaging showed that the median PFS was 12.0 months in the control group but 18.0 months in the bevacizumab-throughout group (HR 0.645, 95 % CI 0.551–0.756, *p* < 0.001).
2. The test for interaction performed in the ICON-7 suggests that the size of the effect of bevacizumab differed between patients at high risk for progression and the rest of the study population (*p* = 0.06). A sub-analysis of patients at high risk of progression defined by stage IV or stage III and suboptimal cytoreduction with residual disease >1 cm showed that the estimated median PFS was 10.5 months with standard therapy, as compared with 16 months with bevacizumab (HR 0.73, 95 % CI 0.60–0.93; *p* = 0.002).
3. In a preliminary overall survival analysis of the ICON-7 requested by regulatory agencies (the number of events is not yet enough for this kind of analysis), it was showed a HR for death in the bevacizumab group of 0.85 (95 % CI 0.69–1.04, *p* = 0.11). However, the test for interaction suggests that the size of the effect of bevacizumab on overall survival differs between the patients at high risk for progression and the rest of the women studied (*p* = 0.011). Among the women at high risk for progression, the median overall survival was 28.8 months in the standard-therapy group and 36.6 months in the bevacizumab group (HR 0.64, 95 % CI 0.48–0.85, *p* = 0.002).

Based on the available data, bevacizumab added to initial chemotherapy followed by a maintenance period of bevacizumab should be deserved for patients who, following standard surgery, are found to have macroscopic residual disease [I, A]. According to exploratory analysis, the benefit seems to be more significant in patients with either stage III disease and residual disease >1 cm, or stage IV disease.

**Fig. 2:**
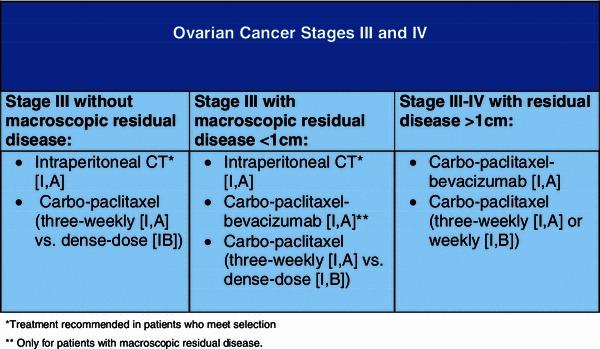
First-line systemic treatment options in advanced ovarian cancer

Figure 2 shows the first-line systemic treatment options in advanced ovarian cancer.

## Therapy for relapsed ovarian cancer

Approximately 70–80 % of patients diagnosed with EOC will suffer a relapse after receiving first-line chemotherapy based on platinum and taxane. According to the Second Consensus on OC held in 1998, a relapse was defined by the presence of at least two of the following criteria [1]:

- Symptoms that may suggest disease (abdominal pain, distension, etc.).
- Clinical or radiological evidence of disease.
- Progressive rise in CA 125, doubly confirmed by GCIG criteria.

A trial by the Medical Research Council and European Organisation for Research and Treatment of Cancer (MRC-OV05) examined the consequences of early institution of treatment for recurrence based exclusively on CA 125 criteria of progression versus treatment delayed until clinical symptoms appeared [89]. The study concluded that there was no survival benefit in the treatment of recurrent OC based exclusively on a rise in the CA 125, and that it anticipates a deterioration in the quality of life [I, A]. In the last Ovarian Cancer Consensus Conference (OCCC), a new classification of recurrent patients was proposed. Distinct patient populations for clinical trial enrolment may be considered according to interval from last platinum therapy. Progression-free interval (PFI) is defined from the last day of platinum until disease progression. The following subgroups should be considered:

- Progression while receiving last line of platinum-based therapy or within 4 weeks of last platinum dose.
- PFI since last line of platinum of <6 months.
- PFI since last line of platinum of 6–12 months.
- PFI since last line of platinum of >12 months.

The authors of this guideline strongly support the use of the classification proposed in the OCCC.

### Treatment of distinct subgroups defined by PFI

Secondary cytoreduction may be appropriate in selected patients despite there is no level 1 evidence which demonstrates a survival advantage. The goal of this surgery is the same as in primary upfront primary surgery. Best candidates for the survival benefit of this surgery are those with a long interval of disease free, no ascites at recurrence, localized disease or few sites of tumor, and complete resection of disease.

For the majority of patients with recurrent EOC, the treatment is based only on systemic therapy. Several factors should be considered in the selection of second-line therapy in EOC:

- Factors depending on the treatment:
  - Response to the last therapy and time since it finished
  - Activity and toxicity of available treatments
  - Ease of administration and cost.
- Factors depending on the patient:
  - Previous and residual toxicity experienced by the patient
  - Clinical condition and previous medical history
  - Preference of the patient.

**Fig. 3:**
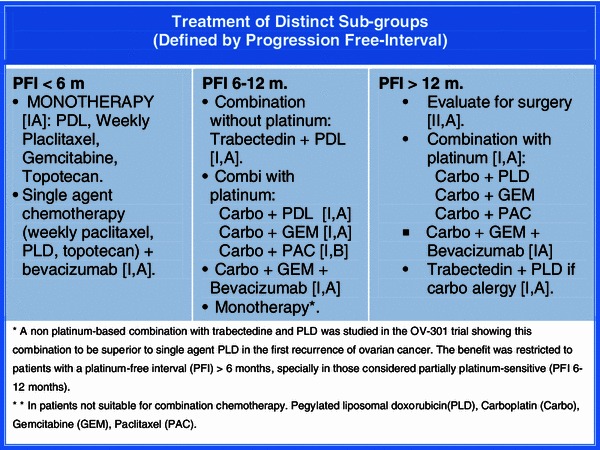
Treatment options in relapsed ovarian cancer. (Defined by Progression Free-Interval)

Figure 3 shows treatment of distinct sub-groups (defined by PFI).

#### Treatment of patients with a PFI <6 months

Patients with platinum-resistant relapse used to be candidates for inclusion in clinical trials with new agents. In the absence of a clinical trial, single-agent therapy without “platinum” is the best palliative option. Several drugs have shown, in phase III trials, some activity with response rates (RR) of 10–15 % and median overall survival (OS) of 9–12 months. Some studies have found combination chemotherapy to be active in patients with a relapse after a PFI <6 months [1]. However, all these studies show that chemotherapy combinations do not improve PFS or OS, but constantly toxicity is significantly higher.

According to these data, in patients with Recurrent OC and a PFI <6 months, sequential single-agent therapy is the best palliative option as quality of life is the most important endpoint [I, A]. A randomized phase III trial evaluating bevacizumab 15 mg/kg every 3 weeks plus chemotherapy versus chemotherapy for platinum-resistant recurrent ovarian cancer [90], provides statistically significant and clinically meaningful improvement in PFS (3.4 vs. 6.7 months), HR 0.48 (0.38–0.60) and objective response rate (ORR) versus chemotherapy alone (30.9 vs. 12.6 %). Strict inclusion criteria minimized the incidence of bevacizumab adverse effects. This is the first phase III trial in platinum-resistant OC to show benefit with a targeted therapy and improved outcome with a combination versus monotherapy [90] [I, A].

#### Treatment of patients with a PFI >12 months

Patients with recurrent disease and a PFI over 12 months are considered fully platinum sensitive, as they use to respond to retreatment with a platinum-based regimen. We have strong evidence, summarized in the Table 8, showing that a platinum-based combination is associated to a longer PFS and also OS in comparison to single-agent platinum chemotherapy [I, A]. As there is no combination that can be considered superior in terms of efficacy, the selection between the different options should be based on the toxicity profile of the different options. Table 8 also summarizes the most relevant toxicities with each combination [91–94].

**Table 8:** Recurrent OC PFI >12 months, two (with platinum) are better than one

Study	*N*	Prior	6–12 Months (%)^a^	Treatment	PFS	HR	95 % CI	OS
		taxane (%)						
ICON 4 [91]	802	43	25	Carboplatin	9 months	0.76	0.66–0.89	24 months
				Carboplatin–Pac	12 months			29 months
GEICO 9801 [92]	81	87.20	42.30	Carboplatin	8.4 months	0.54	0.32–0.92	17 months
				Carboplatin–Pac	12.2 months			–
AGO-EORTC [93]	356	70	40	Carboplatin	5.8 months	0.72	0.58–0.90	17.3 months
				Carboplatin–Gem	8.6 months			18 months
CALYPSO [94]	973	35	99	Carboplatin–Pac	9.4 months	0.821	0.72–0.94	–
				Carboplatin–PLD	11.3 months			

^a^Rate of patients with a platinum-free interval of 6–12 months

*PFS* progression-free survival, *OS* overall survival, *HR* hazard ratio, *95* *% CI* 95 % confidence interval

Hypersensitivity can occur during the second-line treatment. This may occur in up to 25 % of patients; it is more likely with carboplatin and usually appears from the seventh treatment cycle. Reactions may be mild (skin rash) but sometimes are severe (anaphylactic shock). The reintroduction of the drug will depend on the expected benefits weighed against the potential risks. In such a case, one of the desensitizing protocols published in the literature should be followed [1].

A randomized trial of carboplatin–gemcitabine plus bevacizumab or placebo included 484 patients with a recurrent ovarian cancer over 6 months after first line of platinum-based chemotherapy [95]. Patients included should have measurable disease and the primary endpoint was PFS as determined by RECIST progression. The association of bevacizumab increased the median PFS from 8.4 to 12.4 months (HR 0.48, 95 % CI 0.34–0.60) and was confirmed by an independent radiology committee. Additionally, the response rate was also higher (78.5 vs. 57.4 %, *p* < 0.0001). The third pre-planned analysis of overall survival has not shown significant differences (33.4 with bevacizumab vs. 33.7 with placebo), and it has been explained more probably for the long post-progression time and the significant number of chemotherapy lines given during this period. On the basis of these results, bevacizumab was approved for the platinum-sensitive recurrent ovarian cancer by the EMA (European Medicines Agency).

#### Treatment of patients with a PFI 6–12 months

Patients relapsing between 6 and 12 months after the last platinum-based chemotherapy used to have a lower response to platinum than those considered fully platinum sensitive (PFI >12 months) and also a shorter PFS and OS. For this reason, different strategies beyond carboplatin-based regimens are under investigation on this group of patients.

One of these strategies is the use of non-platinum-based regimen, based on the results of the OVA-301 trial [96]. This multinational, multicenter trial assessed the safety and efficacy of trabectedin plus PLD versus PLD alone in platinum-sensitive and platinum-resistant patients with recurrent ovarian cancer. The primary endpoint was PFS. Patients were randomly assigned to receive a 90-min infusion of PLD 50 mg/m^2^ every 4 weeks (*n* = 330) or a 90-min infusion of PLD 30 mg/m^2^ followed by a 3-h infusion of trabectedin 1.1 mg/m^2^ every 3 weeks (*n* = 333). The groups were well matched for baseline characteristics, with 63–65 % of patients classified as platinum sensitive (PFI ≥6 months). The distribution of patients with PFI of 6–12 months and PFI >12 months was fairly equal in these groups. PFS was 7.3 months in patients receiving the combination of PLD and trabectedin versus 5.8 months for patients who received PLD alone (HR 0.79, 95 % CI 0.65–0.96, *p* = 0.0190). Stratifying results according to platinum-resistant or platinum-sensitive status demonstrated that the benefit is observed only in platinum-sensitive patients.

A subgroup analysis showed that the group of patients with a PFI of 6–12 months obtained an increment in OS when treated with trabectedin and PLD than compared to PLD monotherapy [97, 98]. This difference was more evident when platinum was the next regimen used after the progression of the patient to the trial medication, raising the hypothesis that a prolongation of the platinum-free interval by a non-platinum-based regimen could restore the platinum sensitivity and be beneficial for the patient. This hypothesis is the background of the randomized clinical trial INOVATYON (INternational OVArian Cancer Patients Trial With YONdelis), which includes patients with recurrent OC and a PFI of 6–12 months and compares the combination of carboplatin–PLD followed by the regimen selected by the investigator at progression or trabectedin–PLD followed by a platinum-based regimen at progression.

Based on the above-mentioned sub-analysis, the combination of trabectedin and PLD has been proposed as an alternative for patients with a PFI of 6–12 months.

## Strategies for the future in ovarian cancer

The progress in the treatment of OC will be leaded by a better understanding of the biology of the disease and the development of new targeted therapies.

According to the 4th OC Consensus Conference, the most promising areas appear to be the antiangiogenesis and the homologous recombination deficiency [2].

Other promising targets currently being studied based on OC biology include: PI3-kinase and Ras/Raf pathways; folate receptor; and immune targets/cytokines, notch/hedgehog and IGF, which all merit further investigation.

The Consensus also stated three important principles: (1) the necessity of exploring predictive biomarkers to select the adequate patient for the drugs, (2) one of the priorities should be to understand the mechanism of resistance to new drugs, and (3) targeted agents should be studied both as single agents and in combination based on preclinical data [2].

**Fig. 4:**
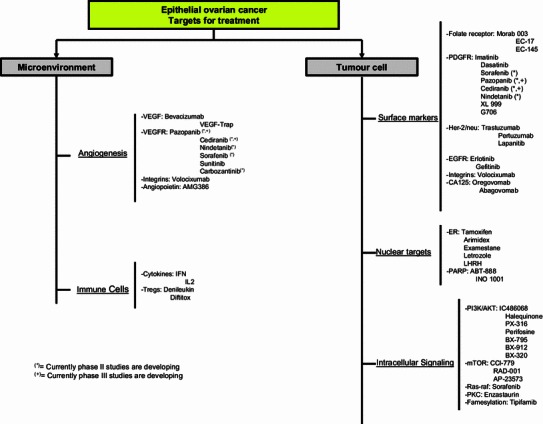
Target for OC treatment based on OC biology

A review of all the targets, the preliminary clinical results and the ongoing clinical trials is beyond the scope of this article. Figure 4 summarizes the main agents.

However, due to the fact that bevacizumab is the only targeted therapy available for clinical use in ovarian cancer right now, it is worth to make some reflections. Specifically, despite the positive results of ICON-7 and GOG-218, several questions remain to be answered about the use of bevacizumab, at least in the front line of ovarian cancer:

- Is PFS a valid end-point or should it be OS? This is an important topic but according to the 4th Ovarian Cancer Consensus Conference, the PFS is the preferred end-point in first line due to the confounding effect of post-progression therapy in ovarian cancer. Nevertheless, OS is still an important endpoint ant it should be maintained in clinical trials when possible.
- Dose selection of bevacizumab? Unfortunately, we do not have an answer for this question, as we have positive results with two different doses (7.5 and 15 mg/kg), without any significant difference in toxicity or a randomized comparison that could help to the clinician.
- Optimal duration of bevacizumab? In both trials, the maximum effect was obtained at the moment when bevacizumab was stopped, and this observation has generated the hypothesis that a longer duration of bevacizumab could be associated to a higher benefit. To explore this interesting clinical question, the AGO group has launched a trial comparing 15 months of bevacizumab in the control arm against 30 months in the experimental arm.
- Other important questions that need to be answered in ongoing and future clinical trials are about the role of bevacizumab in second line when it was used in first line, a well-designed pharmaco-economic analysis and the validation of predictive biomarkers. Fortunately, both first line trials were accompanied by an important translational program seeking for predictive biomarkers on the use of bevacizumab.

## Methodology in clinical trials

A large number of new therapies are being studied in OC, presenting additional challenges, in terms of identifying their activity and their place in the treatment. Establishing optimal treatment as single agent, or in combination with chemotherapy, or as maintenance treatment, requires new approaches to trial design, selection of meaningful endpoints and carefully conducted trials with translational studies.

A clinical endpoint is a characteristic or variable that reflects how a patient feels, functions, or survives, while a surrogate endpoint is a biomarker or endpoint that is intended to substitute for a clinical endpoint. A good correlate may not make a good surrogate. A surrogate endpoint is expected to predict clinical benefit (or harm), or lack thereof [99].

The 2010 Gynecologic Cancer InterGroup (GCIG) consensus statement on clinical trials in OC includes a review of the latest evidence from high-quality clinical trials [2].

Appropriate endpoints for clinical trials should reflect the achievement of clinical benefit, which is defined as improvement of one or more of the following subjective and objective endpoints: toxicity, time without symptoms, patient-reported outcomes (PRO), PFS, overall survival (OS). Cost effectiveness should be evaluated when feasible [2].

We should assess the meaningful endpoints in different OC settings for phase III trials. In adjuvant trials for early disease, RFS (recurrence free survival) is a valid surrogate for OS. In first line for advance disease, PFS is a valid surrogate for OS for trials with chemotherapy. As mentioned before, PFS is the preferred endpoint because of the confounding effect of post-progression therapy. However, when possible, the study should be powered to allow proper assessment of both PFS and OS. Finally, how the PFS is defined should be established with regard to method (CA-125; RECIST) and timing of evaluation.

In platinum-sensitive relapse, if PFS is used as primary endpoint, trials should be powered for OS as co-primary endpoint, otherwise OS is the preferred primary endpoint for phase III trials [2].

In resistant-refractory, we will consider composite endpoints involving QoL aspects due to clinical relevance of the patients [2]. Secondary endpoints may include objective response rate, percent survival at 6 months, health-related quality of life, PRO, time without symptoms or toxicity, and pharmacoeconomic analyses. PFS is the preferred endpoint (over ORR at a time point) and ORR is not a validated endpoint when testing new agents.

The conference addresses a number of molecular markers as surrogate outcomes and predictive factors; however, although most of these markers hold significant promise, there is no standard molecular profile that must be included in all trials. It was recommended that the collection of biological specimens be considered in each and every clinical trial at predetermined intervals. This recognizes that there are multiple issues to be addressed and harmonized in the collection of tissues in different jurisdictions and central analysis [2].

## Cooperation in research in Gynecological Oncology

To organize, stimulate and coordinate clinical research studies on gynecological cancer in general, but especially on ovarian cancer, multidisciplinary specialist networks need to be set up which can then form working subgroups with the participation of oncologists, surgeons, pathologists, pharmacoeconomic specialists, epidemiologists, etc.

Society meetings should promote academic clinical trials, disseminate the results of important trials, and facilitate the development and coordination of new trials. Finally, the leadership of our sister societies should meet several times a year to share information and coordinate educational programmes. We must continue to work together to reduce the global burden of gynecological cancer.
